# Influence of Urea and Dimethyl Sulfoxide on K-Peptide Fibrillation

**DOI:** 10.3390/ijms23063027

**Published:** 2022-03-11

**Authors:** Jarosław Wawer, Jakub Karczewski, Robert Aranowski, Rafał Piątek, Danuta Augustin-Nowacka, Piotr Bruździak

**Affiliations:** 1 Department of Physical Chemistry, Faculty of Chemistry, Gdańsk University of Technology, Narutowicza Str. 11/12, 80-233 Gdańsk, Poland; piotr.bruzdziak@pg.edu.pl; 2 Institute of Nanotechnology and Materials Science, Faculty of Applied Physics and Mathematics, Gdańsk University of Technology, Narutowicza Str. 11/12, 80-233 Gdańsk, Poland; jakub.karczewski@pg.edu.pl; 3 Department of Process Engineering and Chemical Technology, Gdańsk University of Technology, Narutowicza Str. 11/12, 80-233 Gdańsk, Poland; robert.aranowski@pg.edu.pl; 4 Department of Molecular Biotechnology and Microbiology, Gdańsk University of Technology, Narutowicza Str. 11/12, 80-233 Gdańsk, Poland; rafpiate@pg.edu.pl; 5 BioTechMed Center, Gdańsk University of Technology, Narutowicza Str. 11/12, 80-233 Gdańsk, Poland; 6 Faculty of Chemistry, University of Gdańsk, Wita Stwosza Str. 63, 80-308 Gdańsk, Poland; d.augustin.nowacka@gmail.com

**Keywords:** amyloids, fibrillation, K-peptide, hen egg white lysozyme, urea, dimethyl sulfoxide

## Abstract

Protein fibrillation leads to formation of amyloids—linear aggregates that are hallmarks of many serious diseases, including Alzheimer’s and Parkinson’s diseases. In this work, we investigate the fibrillation of a short peptide (K-peptide) from the amyloidogenic core of hen egg white lysozyme in the presence of dimethyl sulfoxide or urea. During the studies, a variety of spectroscopic methods were used: fluorescence spectroscopy and the Thioflavin T assay, circular dichroism, Fourier-transform infrared spectroscopy, optical density measurements, dynamic light scattering and intrinsic fluorescence. Additionally, the presence of amyloids was confirmed by atomic force microscopy. The obtained results show that the K-peptide is highly prone to form fibrillar aggregates. The measurements also confirm the weak impact of dimethyl sulfoxide on peptide fibrillation and distinct influence of urea. We believe that the K-peptide has higher amyloidogenic propensity than the whole protein, i.e., hen egg white lysozyme, most likely due to the lack of the first step of amyloidogenesis—partial unfolding of the native structure. Urea influences the second step of K-peptide amyloidogenesis, i.e., folding into amyloids.

## 1. Introduction

Amyloids are fibrillar aggregates of proteins. The growing interest in the subject of protein fibrillation results from its medical importance. Around 40 serious and often fatal disorders in humans are associated with the formation of amyloids [1]. A characteristic feature of amyloids is their morphological structure. They are unbranched and long, with diameter from 2 to 12 nm [2,3]. The secondary structure is rich in β-sheets [4] and the monomeric protein molecules are held together by non-covalent bonds [5].

The ability to form amyloids is not unique to large proteins; shorter peptides are also capable of this type of aggregation. The most well-known amyloidogenic peptides are probably: amyloid beta [6] and islet amyloid polypeptide [7]. These peptides form the pathological structures and are involved in the development of Alzheimer’s disease and type 2 diabetes, respectively. Despite the negative aspects of amyloidogenesis, the fibrillar aggregates of peptides are considered interesting starting materials with a number of prospective applications. It was reported that they may be used for synthesis of the compounds useful in: bio-imaging [8], tissue engineering [9], construction of novel biosensors [10], formation of hybrid nanocomposites for electronics [11], as catalysts in organic synthesis [12] and as nanovaccines [13]. The research on peptide fibrillation can not only provide insight into protein misfolding relevant to the biological systems but also be useful in technical applications.

Spectroscopic methods are the most basic tools used to study protein fibrillation. In the current work, we utilized a variety of spectroscopic techniques which were sensitive to different aspects of amyloidogenesis.

Fluorescence spectroscopy with the Thioflavin T (ThT) assay is regarded as a standard method toward preliminary detection of amyloid fibrils [14,15,16]. We used it to estimate the amount of amyloids in the investigated samples. Circular dichroism in the UV region (CD) [17,18,19,20] and Fourier-transform infrared spectroscopy (FTIR) [21] provide information about the secondary structure of protein. Both methods have their limitations, but—due to their sensitivity to different physical processes—they, to some point, complement each other. This fact was exploited in our research. UV-VIS spectroscopy and optical density measurements (OD) allow for assessment of overall aggregation capacity. The recorded absorbance depends simultaneously on size and number of aggregates. The analysis of the size distribution of formed aggregates can be further performed by means of dynamic light scattering (DLS). Intrinsic fluorescence helps in obtaining data on the immediate surroundings of tryptophan (Trp). The change of the intensity of Trp fluorescence and the shift of maximum of the spectrum are directly related to access of these residues to water and polarity of the microenvironment [22]. The changes around tryptophan reflect the spatial rearrangements of the polypeptide chain and protein aggregation.

The results of spectroscopic methods must necessarily be confronted with imaging using microscopic techniques, such as transmission electron microscopy or atomic force microscopy (AFM). These measurements allows for detection of false negative results [23]. By applying all of the above-mentioned techniques, it is possible to obtain a comprehensive picture of fibrillation.

The aim of the present study was to investigate fibrillation of the K-peptide in solutions of various compositions. The K-peptide (GILQINSRW) is a fragment—residues 54–62—of the hen egg white lysozyme (HEWL). It was identified by Yasushi Sugimoto et al. in 2011 [24] as a core for the amyloidogenesis of HEWL [25]. We tested the influence of dimethyl sulfoxide (DMSO) and urea on amyloidogenesis. Dimethyl sulfoxide is a solvent widely used to improve the solubility of compounds in fibrillation-related studies, but research on its impact on amyloid fibril formation is rarely conducted [26,27,28]. Urea is a well-characterized mild protein destabilizer [29]. In our earlier work [30], we investigated the influence of denaturants on fibrillation, but the study was conducted for the whole HEWL protein. In the present work, we chose a concentration of additives to obtain data which could be related to our previous studies [30,31] but high enough to observe the influence of the investigated compounds on amyloidogenesis.

The results of the current work show that impact of the protein destabilizer is different for the K-peptide than for HEWL. We demonstrate that urea affects the mechanism of fibrillation by targeting—in the case of the short peptide—the second step of the amyloidogenesis, i.e., the folding of the peptide into amyloids. Moreover, we speculate that the high amyloidogenic propensity of the K-peptide may indicate that the short peptides play a certain role in amyloidogenesis of whole proteins.

## 2. Materials and Methods

### 2.1. Solution and Fibril Preparation

K-peptide with amino acid sequence GILQINSRW was purchased from Lipopharm.pl, Zblewo, Poland (purity > 95%). The mass spectra and high performance liquid chromatography profile are presented in the Supplementary Materials. The calculated parameters for peptide are as follows: pI = 9.75 (Swiss Institute of Bioinformatics Expasy service), Log P = −4.54 [32]. The peptide was dissolved in 20 mM acetate buffer, pH = 4, in the presence of additives, i.e., dimethyl sulfoxide (Alfa Aesar, Thermo Fisher GmbH, Kandel, Germany, purity ≥ 99.9% ) or urea (VWR, Leuven, Belgium, purity ≥ 99.5%). The samples were gently mixed for 2 h, at room temperature, to completely dissolve the peptide. Concentration of the K-peptide was close to saturation point in water and was equal to 2 mg/mL. The concentration of the used additives was equal to 0.5 M. The solutions of K-peptide in the buffer without additives and in the pure water were also prepared. All samples were incubated in a water bath (Grant LSB AquaPro) at 37 °C for 14 days. The obtained solutions were diluted when necessary and analysed using various spectroscopic methods.

### 2.2. Thioflavin T (ThT) Fluorescence Assay

The 2.5 mM solution of Thioflavin T (Sigma-Aldrich Cat. No. T3516, Steinhein, Germany) was prepared in 50 mM phosphate buffer, pH = 6.7. The ThT stock solution and the incubated samples were mixed with 50 mM phosphate buffer, pH = 6.7, and the final concentration of the working solution was equal to 0.12 mg/mL for K-peptide and 25 μM for ThT. In order to prepare the samples for analysis, the following were mixed together: 250 μL of K-peptide solution, 40 μL of the ThT stock solution and 3800 μL of buffer. After the preparation, the samples were stored at room temperature for one hour. The fluorescence spectra were recorded using JASCO FP 8300 Spectrofluorometer. The excitation wavelength was set to 445 nm and the emission spectra were recorded in the range of 460 to 550 nm. The photomultiplier voltage was equal to 400 V and the scan speed was equal to 200 nm/min. The excitation and emission bandwidths were set to 5 and 10 nm, respectively. At least 5 scans were averaged for each sample.

### 2.3. Atomic Force Microscopy (AFM)

The K-peptide solutions were diluted with deionized and filtered water (syringe filters 0.1 μm, polyethersulfone membrane) to the concentration of 0.2 mg/mL. Fifty μL of this solution was placed on a freshly cleaved surface of mica plate. After 5 minutes of incubation, mica plates were washed with two portions (50 μL each) of the filtered water. The plates were dried and stored in a desiccator over silica gel. The scans of the surface were collected using Nanosurf Easyscan 2 microscope in the contact mode. The scan speed was set to 1.2–1.4 s per line, and the applied force was equal to 12–15 nN. The resolution of the scans was 1024 × 1024 px and the scans covered an area of 9 × 9 μm. The data were analysed in Gwyddion 2.50.

### 2.4. Optical Density Measurements (OD)

The concentration of the K-peptide in the analysed samples was equal to 2 mg/mL. The optical density of the samples was measured using Thermo Scientific Evolution 300 Spectrophotometer at 350 nm. The optical length was equal to 1 cm. The absorbances of the buffer and buffer with additives were not subtracted from the measured optical density, as it was not necessary. Ten scans were collected for each sample and averaged.

### 2.5. Dynamic Light Scattering (DLS)

The K-peptide solutions with concentrations of 2 mg/mL were used for analysis. The samples were placed in disposable cuvettes with optical lengths of 1 cm. Dynamic light scattering was measured using Malvern Zetasizer Nano ZS. The wavelength of the laser was equal to 633 nm. The radiation was collected in the backscatter mode (angle 173°). Each scan was measured for 20 seconds and, for a single determination, 20 runs were accumulated. Three scans obtained in this way were averaged for each sample. Temperature of the compartment was equal to 25 °C. The standard set of parameters were used in data analysis. The values 1.450 and 0.001 were accepted as the refractive index of material and the absorption, respectively. For dispersant, a viscosity of 0.8872 cP and refractive index of 1.330 were taken. The particle size distributions were obtained using non-negative least squares method (NNLS). The correlation functions for dynamic light scattering measurements are shown in Supplementary Materials in Figure S1.

### 2.6. Circular Dichroism Spectroscopy (CD)

Circular dichroism spectra, in mdeg, were collected using JASCO J-815 Spectropolarimeter. Before measurements, samples were diluted with water to the finial concentration of the K-peptide in the range of 0.1–0.07 mg/mL. The exact concentration of the peptide was determined spectrophotometrically (see Section 2.7). Quartz cell with optical length equal to 0.1 cm was filled with solution. Data points were recorded with 0.2 nm intervals, and scan speed was set to 50 nm/min. The signals measured for the blank samples without the K-peptide were subtracted from the spectra recorded for solutions containing the peptide. Six scans were averaged for each sample. The ranges of high-tension (HT) voltage applied to the photomultiplier in circular dichroism measurements are shown in Supplementary Materials in Table S1.

The obtained data were analysed with the help of the DichroWeb on-line service [18,33]. The CDSSTR algorithm was used with the reference data set 7 [34].

### 2.7. UV-VIS Absorbance Spectroscopy

In order to interpret circular dichroism data, it is essential to know the exact concentration of the peptide in a sample. To achieve this goal, the part of solution used for the CD measurements was analysed by means of UV-VIS spectroscopy. The spectra were collected using Thermo Scientific Evolution 300 Spectrophotometer. The absorbances were measured at 280 nm. The extinction coefficient was theoretically estimated as ϵ = 5.063 dm^3^g^−1^cm^−1^ using Swiss Institute of Bioinformatics Expasy service. For each sample, three scans were averaged.

### 2.8. Infrared Spectroscopy (FTIR)

The undiluted solutions of the K-peptide (2 mg/mL) were used for the Fourier-transform infrared spectroscopy measurements. The spectra were collected using Nicolet 8700 FTIR Spectrometer equipped with EverGlo IR source, KBr beamsplitter and DTGS-TEC detector (Thermo Scientific, Waltham, MA, USA). The Golden Gate ATR accessory (Specac) equipped with a single reflection diamond crystal was used. The 128 independent scans were averaged for each sample with the resolution set to 2 cm^−1^ and 1 cm^−1^ data spacing. The samples of the K-peptide were dried on the ATR crystal. The spectrophotometer was purged with dry nitrogen to remove the water vapour contamination. The data were analysed using Omnic software. Each spectrum was corrected by subtracting atmosphere spectra, measured a few times between samples. The second derivatives of absorbance were calculated using the Savitsky–Golay algorithm (13 points, 3rd order polynomial).

### 2.9. Intrinsic Fluorescence of Tryptophan Residues

The solution of K-peptide (2 mg/mL) was placed into quartz cuvette (light path 10 mm × 10 mm with four polished windows) and the fluorescence spectra were recorded using JASCO FP 8300 Spectrofluorometer. The samples were excited at 280 nm, and the excitation and emission bandwidths were set to 5 nm. Scan speed was equal to 1000 nm/min, and the photomultiplier voltage was set to 450 V. The emission spectra were recorded in the range of 300 to 460 nm with an interval of 0.5 nm. Three scans were averaged for each sample.

## 3. Results and Discussion

### 3.1. Detection of Amyloids by Thioflavin T Fluorescence Assay

**Figure 1:**
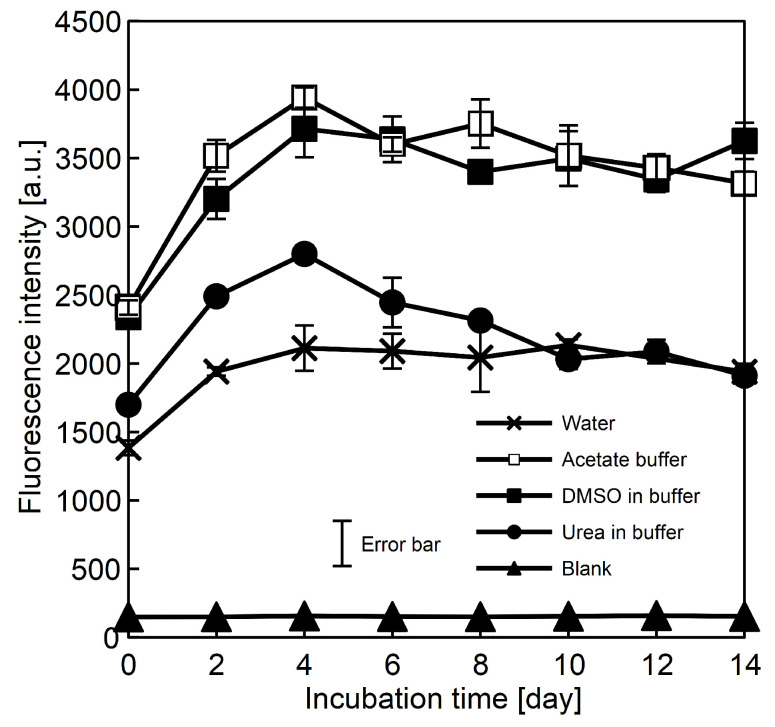
The ThT florescence intensity recorded at 485 nm for the samples of K-peptide incubated at 37 °C up to 14 days. The peptide was dissolved in: ✕ water; □ acetate buffer, pH = 4, without additives; ■ acetate buffer with dimethyl sulfoxide and ● acetate buffer with urea. Symbol ▲ refers to blank, control solutions without the K-peptide. Experimental uncertainty is presented as standard deviation.

The ThT fluorescence assay is a method used in preliminary detection of amyloids [14,15,16]. We conducted the measurements for the initial, freshly prepared solutions and for the samples incubated up to 14 days. The obtained results for all systems are presented in Figure 1.

As can be seen, starting fluorescence for all of the tested samples was significantly higher than for the control solution. This fact suggests that the peptide undergoes fibrillation but the ThT test alone does not prove that the solutions contain amyloids. However, the measurements performed by us, i.e., by atomic force microscopy, circular dichroism spectroscopy and infrared spectroscopy, strongly support the conclusion that amyloids were present in the analysed samples (see Section 3.2, Section 3.5 and Section 3.6).

It seems that the amyloids were formed very quickly after dissolution, and after 4 days of incubation at 37 °C, the signal reached plateau. The concentration of the amyloids after this period of time was maximal.

The lowest signal of the ThT fluorescence was detected for samples dissolved in water and for acetate buffer with urea. The low signal for the K-peptide in water solution can be attributed to the acidity of this sample. It has been previously shown [25] that the fibrillation efficiency depends on pH of the solution, and for the studied peptide, efficiency was the highest at around pH = 4. The solution of K-peptide in pure water has pH = 3.1, which might be slightly outside of the optimal range. In the case of the urea solution, the amount of fibrils was markedly lowered in comparison to other samples at pH = 4. For the urea solution, the highest fluorescence intensity was recorded after four days of incubation; after which, the signal decreased. This signifies that urea disturbs the fibrillation of K-peptide. The experimental data show that presence of DMSO in the buffer solution exerts negligible impact on K-peptide fibrillation.

### 3.2. The Micrographic Structure of K-Peptide Aggregates

**Figure 2:**
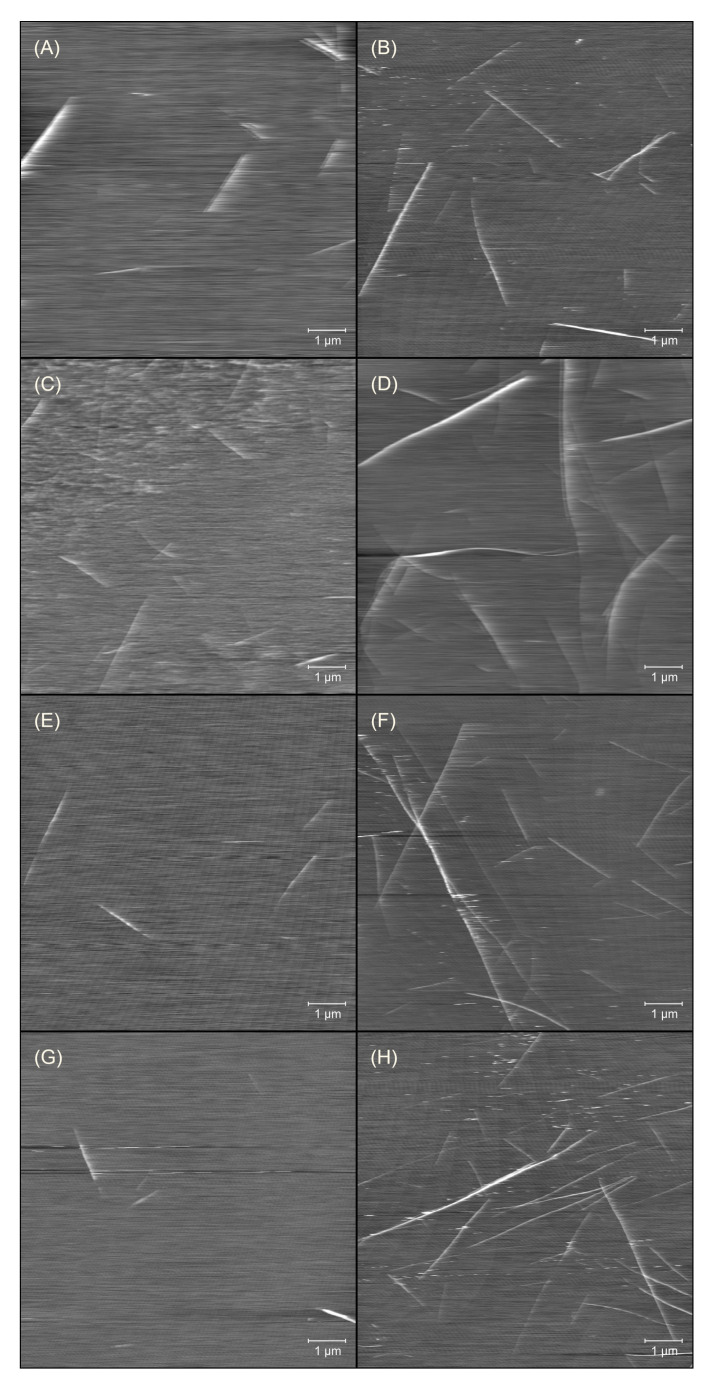
The atomic force microscopy scans for the samples of K-peptide in freshly prepared solutions (left column) and in the samples incubated at 37 °C for 14 days (right column). The peptide was dissolved in: (**A**,**B**) water; (**C**,**D**) acetate buffer, pH = 4, without additives; (**E**,**F**) acetate buffer with dimethyl sulfoxide and (**G**,**H**) acetate buffer with urea.

The presence of amyloids in analysed samples was confirmed by means of atomic force microscopy (AFM). The recorded scans are presented in Figure 2. As expected from the ThT measurements, the amyloids were detected in non-incubated samples. The AFM showed that the number of fibrils increased during prolonged heating.

It appears that the amyloid concentration was the lowest for the freshly prepared solution containing urea and, among the incubated samples, for the K-peptide solutions in pure water. However, in the present study, atomic force microscopy was not used to quantify the number of fibrils but only for visualization purposes. This first aim was achieved with higher accuracy with the help of the measurements of ThT fluorescence.

The amyloids in all non-incubated samples (except the sample containing urea) were 1.5 μm long, sporadically reaching 3 μm. The height of fibrils was around 2.5 nm, sporadically, 5 nm. In the presence of urea, amyloids were of similar thickness but shorter, around 1 μm.

After incubation, the amyloids—formed in the solution of the K-peptide in pure water—were significantly longer, typically around 2.5–4 μm, and thicker, 4–12 nm, than the unheated samples. The lengths of fibrils generated in other solutions were more diverse. The amyloids, 1.5 μm long, were present as well as very long fibrils, longer than 8 μm. The amyloids in these samples seemed to be thinner than the fibrils formed in pure water and were 3–8 nm high.

The parameters given above should be understood as a rough estimation of the fibrils’ dimensions. The AFM scan depends strongly on the place where the scan was performed. For this reason, and due to the at least partially subjective assessment, the given values should be considered approximate.

The striking feature of the K-peptide fibrils’ morphology is that the obtained amyloids are needle-like and stiff. The amyloids of the whole protein—hen egg white lysozyme—tend to be more curvy and elastic [23,35].

### 3.3. Determination of Optical Density of Samples

To assess the progress of aggregation of K-peptide, the optical density measurements were performed. The obtained results are presented in Table 1.

The highest signals were recorded for the K-peptide in acetate buffer and acetate buffer with DMSO after 14 days of incubation. The lowest signals corresponded to freshly prepared solutions of the K-peptide in pure water and acetate buffer with urea. These results are in agreement with that of ThT assay. Both of the measured parameters—optical density and ThT fluorescence intensity—increased during incubation. The rise of the OD signal was about 40–60%.

However, there is an important observation that needs to be commented upon. The highest percentage increase in the sample optical density was recorded for the solution containing urea. A closer inspection of Figure 1 reveals that, although initially, the ThT signal increased for this sample, after 4 days of incubation, the signal reached maximum and begin to decrease to a final value very close to the starting value. This fact, together with OD data, indicates that the aggregation of K-peptide in the presence of urea is occurring but does not necessarily lead to the formation of amyloids. In the case of this sample, the amorphous aggregates were also formed. It should be noticed that urea does not drastically increase the aggregation. In comparison to the other samples, the absolute value of OD signal is relatively low. The observations described above can serve as strong evidence that urea influences K-peptide fibrillation.

### 3.4. Size Distribution of Aggregates Analysed by DLS

**Figure 3:**
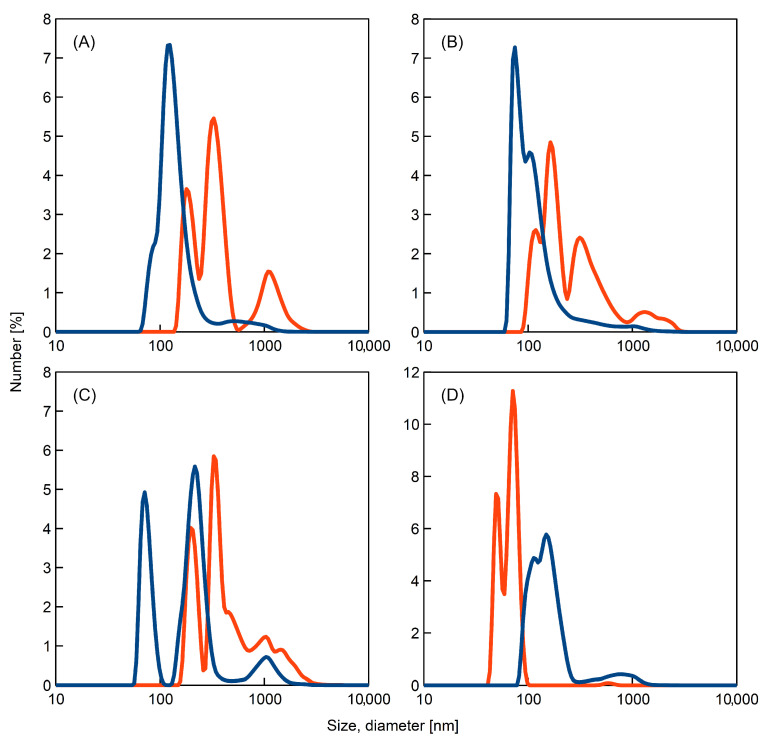
Size distribution by number obtained from dynamic light scattering measurements for the samples of K-peptide in freshly prepared solutions (dark blue line) and for the samples of K-peptide incubated at 37 °C for 14 days (red line) dissolved in: (**A**) water; (**B**) acetate buffer, pH = 4, without additives; (**C**) acetate buffer with dimethyl sulfoxide and (**D**) acetate buffer with urea.

The analysis of the size distribution of the aggregates was performed by means of dynamic light scattering (Figure 3). This method is preferably used for globular aggregates but it can be also applied for the samples containing fibrils [3,19].

In general, the incubation of samples moved size distribution to higher values of aggregate size. Additionally, the intensity of the band, about 1000 nm, increased. These observations are in agreement with AFM measurements; incubation leads to the formation of longer fibrils.

The results for samples containing urea are clearly different. Surprisingly, heating of the solution of K-peptide in the presence of this additive shifted its size distribution to lower values. This observation can be linked with the decrease in the ThT signal after reaching maximum fluorescence. The presented DLS plot for incubated samples of K-peptide with urea suggests that this solution is fairly homogeneous. In comparison to the other samples, the recorded peak is relatively narrow and high. Optical density measurements and ThT assay, along with the AFM imaging supplemented with DLS results, provides consistent interpretation of data for this sample. After incubation of K-peptide in the urea solution, apart from the long fibrils, a large number of smaller but amorphous aggregates were formed. It is possible that during incubation, additional processes occurred for this experimental system.

### 3.5. Secondary Structure Changes Monitored by Circular Dichroism Spectroscopy

**Figure 4:**
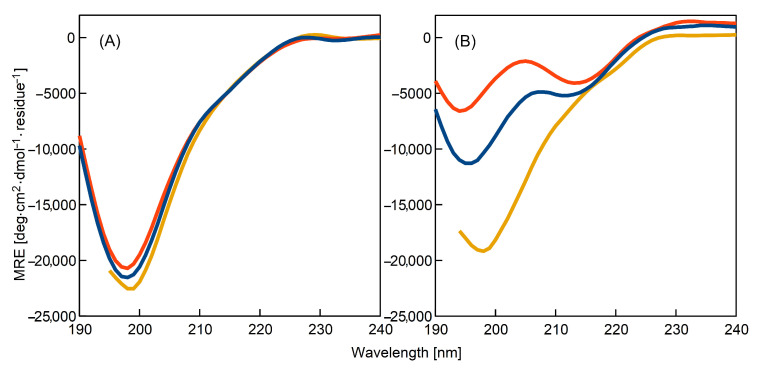
Mean residue ellipticity (MRE) recorded for: (**A**) samples of non-incubated freshly prepared solutions of the K-peptide, (**B**) samples of the K-peptide incubated at 37 °C for 14 days. The peptide was dissolved in: water (blue line); acetate buffer, pH = 4, without additives (red line) and acetate buffer with urea (dark yellow line).

To investigate the changes of the secondary structure of the K-peptide during incubation, we performed the circular dichroism measurements in the UV region. The obtained spectra are presented in Figure 4.

Unfortunately, it was impossible to record the signal for the solutions containing DMSO due to their high absorbance. We analysed these solutions using a complementary technique, i.e., Fourier-transform infrared spectroscopy (see Section 3.6).

Inspection of Figure 4 reveals that incubation significantly changes the intensity and shape of the mean residue ellipticity (MRE). The minimum, at 198 nm, diminished and, for the K-peptide dissolved in water and acetate buffer without additives, moved slightly to the lower wavelengths—195 nm. For both of the mentioned samples, a new minimum at around 212 nm emerged. These changes can be interpreted as evidence of the reduction in irregular structures and increase in the β-sheet content in the K-peptide’s spatial configuration [17]. The highest change was recorded for the K-peptide dissolved in acetate buffer, although the transformation for the water solution was also significant.

In the case of the sample containing urea, the shape of the spectrum remained mostly unaffected. The decrease in MRE for incubated solution was only marginal in comparison to that of the unheated sample. This observation serves as additional strong evidence showing significant influence of this additive on K-peptide fibrillation and its inhibitory action against amyloidogenesis. The close agreement of the CD measurements with ThT fluorescence data is noteworthy. After the initial rise of the ThT intensity, the signal declined to the starting value of ThT fluorescence.

The collected CD data for the samples of K-peptide in the water and acetate buffer without additives allowed us to calculate the percentage of secondary structure components [18,33,34]. The obtained results are shown in Table 2.

The structure of the K-peptide in freshly prepared samples was devoid of the α-helices and highly unstructured. The percentage of disordered components was close to 80%. The β-sheets were present and constituted 17–19% of the structure. The content of β-sheets was somewhat higher for the K-peptide in acetate buffer without additives than for the K-peptide dissolved in water.

The comparison of the non-incubated and incubated samples leads to an interesting observation The content of the unordered structures decreased after heating. For the K-peptide in water solution, this decrease was rather moderate (–11%), but for the K-peptide in acetate buffer, it was significantly higher (–20%). The β-sheet content increased for both types of samples. However, the β-structures’ increase was higher for the K-peptide in acetate buffer than for the K-peptide in water solution. The final percentage of the β-structures for the sample containing acetate buffer was fairly large (39%).

In summary, we note that CD results are in good agreement with data obtained from ThT fluorescence analysis. The β-sheets were present in the freshly prepared samples and the content of the β-assemblies increased during incubation. The presence of urea inhibited amyloidogenesis.

### 3.6. Secondary Structure Changes Monitored by Infrared Spectroscopy

**Figure 5:**
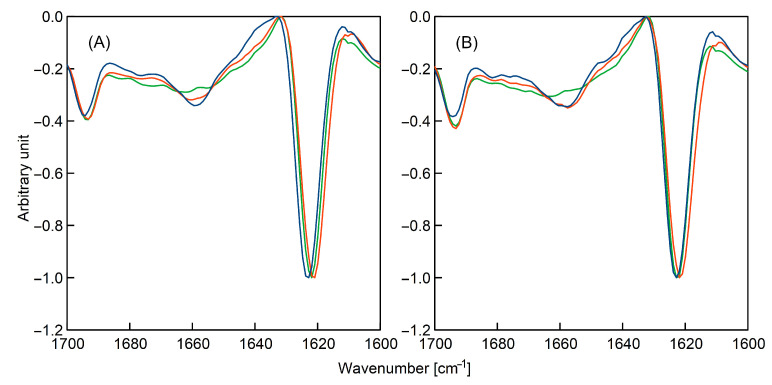
Infrared spectra—expressed as second derivatives of absorbance—recorded for: (**A**) samples of non-incubated freshly prepared solutions of the K-peptide and (**B**) samples of the K-peptide incubated at 37 °C for 14 days. The peptide was dissolved in: water (blue line); acetate buffer, pH = 4, without additives (red line) and acetate buffer with dimethyl sulfoxide (green line).

As has been mentioned above, the samples with DMSO could not be analysed using circular dichroism; thus, infrared spectroscopy measurements were conducted. The measured FTIR spectra—expressed as second derivatives of absorbance vs. wavenumber—are presented in Figure 5. In the Supplementary Materials, we also show the IR spectra (Figure S2, normalised to the common maximum intensity of the amide I band) and graphs useful for analysing the effect of incubation on IR spectra (Figure S3).

Unfortunately—due to the overlapping of bands—FTIR did not allow us to examine the samples containing urea; however, circular dichroism provided insight into the influence of this additive on K-peptide fibrillation (see Section 3.5). As can be seen, both techniques are, to some point, supplementary and, in this way, it is possible to obtain information about the conformation of the amino-acid chain.

The analysed region, 1700–1600 cm^−1^, corresponds to the amide I band and is sensitive to changes in the secondary structure of the peptide [21,36]. To simplify the interpretation, the second derivatives of the IR spectra were calculated [37] and the obtained data were normalized to common maximum.

As can be seen, in all samples, the band at around 1623 cm^−1^ dominated. A smaller but noticeable peak was present at 1693 cm^−1^. The occurrence of these two characteristic absorption regions indicates a strong presence of β-structures [38,39,40].

In the case of unheated samples, only a minor difference can be noticed between obtained spectra. The spectrum of the K-peptide in the water solution deviated slightly from the rest of the data. During incubation, the spectra became more similar to each other. The infrared spectroscopy measurements confirm the findings obtained using other experimental techniques. The β-sheet structures were present in the starting solutions. The IR spectra show that the structure of the K-peptide is similar in the solutions of acetate buffer and acetate buffer with DMSO.

### 3.7. Structural Transformations Detected by Intrinsic Fluorescence of the K-Peptide

**Figure 6:**
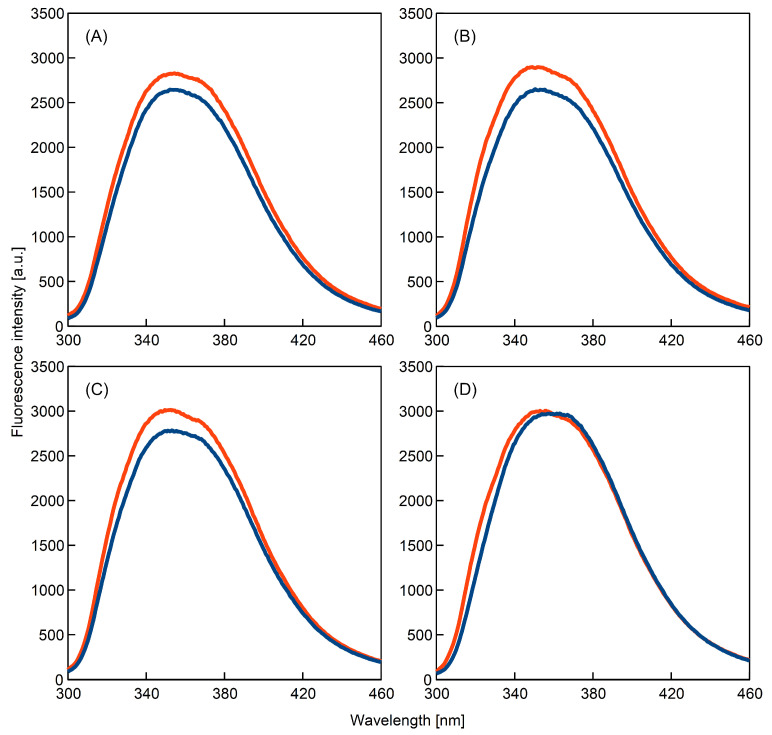
Intrinsic fluorescence of the K-peptide in freshly prepared solutions (dark blue line) and its fluorescence after incubation at 37 °C for 14 days (red line). The peptide was dissolved in: (**A**) water; (**B**) acetate buffer, pH = 4, without additives; (**C**) acetate buffer with dimethyl sulfoxide and (**D**) acetate buffer with urea.

Rearrangement of the three-dimensional structure of the K-peptide was monitored by the measurement of intrinsic fluorescence. Tryptophan residues located at the end of the amino-acid chain make this kind of research possible. In the present work, we compared the fluorescence spectra obtained for freshly prepared solutions with the data collected for samples incubated for 14 days (Figure 6).

The fluorescence of the Trp depends on the character of the local environment around this residue [22]. It was suggested that aromatic residues play important roles in amyloidogenesis [41], and thus, it can be expected that Trp fluorescence measurements provide valuable insight to fibrillation mechanisms.

The experimental data obtained by us show that the samples’ incubation leads to increases in fluorescence intensity for all samples except the urea solution. At the acidic pH, this increase can be regarded as a sign of ongoing fibrillation [42].

The intensity of fluorescence is often related to the static and dynamic quenching of Trp by the solvent molecules [22]. The increase detected by us indicates that tryptophans become less accessible to solvent molecules and Trp quenching is reduced. This phenomenon is another consequence of the ongoing K-peptide aggregation.

In contrast to the other samples, the fluorescence spectrum recorded for the K-peptide solutions containing urea remains almost unaffected. Under the conditions of our experiments, this additive—to a significant extent—exhibits inhibitory activity against amyloidogenesis.

For the samples with urea, only the minor blue shift was observed. It was suggested in literature [22] that the position of the maximum of fluorescence intensity spectrum depends on the polarity of the microenvironment around Trp. The shift of the maximum towards lower wavelengths may result from the transition of Trp to more non-polar environments environment [22]. The change recorded by us is, however, rather small.

The fact that fluorescence spectrum for the K-peptide in the urea solution incubated for 14 days is roughly the same as the spectrum for the freshly prepared solution is in particularly good agreement with the conclusions drawn from CD and ThT analysis.

## 4. Conclusions

The current work sheds new light on the fibrillation mechanism. The spectroscopic techniques and atomic force microscopy lead to a consistent picture of K-peptide aggregation dynamics.

The experimental results presented in the current work show that DMSO has little influence on K-peptide fibrillation. It is important information from a practical point of view because many research procedures include the dissolution of peptides [25] or additives [43,44] in this solvent. In this manner, the problems with low solubility of compounds can be avoided, but, unfortunately, studies on impact of the DMSO on amyloidogenesis are rarely conducted [26].

Urea is a well-known protein destabilizer [29,45]. Our earlier work [30], in which we investigated influence of denaturants on full lysozyme protein fibrillation, revealed that the addition of protein destabilizer may increase efficiency of amyloidogenesis. The results of our current paper show that K-peptide fibrillation is affected in a different manner. The influence of this additive may be explained with the help of a classical approach, according to which amyloidogenesis can be regarded as a complex set of transformations. Most importantly, two distinct steps can be identified: (i) partial unfolding of the macromolecule and (ii) folding into amyloids [46]. In the case of large proteins, destabilizers promote the first step of amyloid fibril formation, i.e., partial unfolding, and thus increase the efficiency of fibrillation. The K-peptide is too short to have a complex ternary structure. Therefore, the first step is not present and the fibrillation is only limited by the second step, i.e., folding into amyloids. Under the experimental conditions of our work, urea manifests its destabilizing properties, modulates the second step and decreases the fibrillation efficiency. It is worth noticing that this observation may be especially important for the fibrillation of intrinsically disordered peptides and proteins (amyloid beta and alpha-synuclein) [6,47].

The results of current study suggest that a short peptide may have higher fibrillation propensity than the whole protein. The reason behind this difference lies probably in the absence of the partial unfolding step. The conclusion drawn here is supported by earlier reports [25,48,49], which also underline the high tendency of fragments of hen egg white lysozyme to form amyloids. This observation is of practical importance and may be useful for the planning of experiments. The factors that increase the fibrillation of whole protein, e.g., high temperature or ionic strength, may not necessary be optimal for the fibrillation of the peptide.

Due to the high amyloid propensity of the short peptides, their role in the regulation of whole protein fibrillation may be considerable but often underestimated. This aspect of amyloidogenesis was investigated before [48,50], but, in our opinion, it is worth further study. 

## Figures and Tables

**Table 1:** The optical densities recorded at 350 nm for freshly prepared solutions of the K-peptide and for the samples incubated at 37 °C for 14 days.

	Optical Density for Sample Incubated for:	
Samples	0 Days	14 Days
K-peptide in water	0.1838	0.2772
K-peptide in acetate buffer	0.2336	0.3279
K-peptide in acetate buffer with DMSO	0.2227	0.3140
K-peptide in acetate buffer with urea	0.1827	0.2900

**Table 2:** The calculated components of the secondary structure for the samples of K-peptide in freshly prepared solutions and for samples incubated at 37 °C for 14 days. Calculation method: CDSSTR algorithm with the reference data set 7 [18,33,34].

Incubation Time	α-Helix [%]	β-Sheet [%]	Turns + Unordered [%]
Samples of K-peptide in water			
day 0	1	17	81
day 14	0	29	70
Samples of K-peptide in 20 mM acetate buffer, pH = 4, without additives			
day 0	1	19	79
day 14	0	39	59

## Data Availability

The data presented in this study are available on request.

## Supplementary Materials

The following supporting information can be downloaded at: https://www.mdpi.com/article/10.3390/ijms23063027/s1.

## Abbreviations

The following abbreviations are used in this manuscript:

AFM	Atomic force microscopy
CD	Circular dichroism spectroscopy
DLS	Dynamic light scattering
DMSO	Dimethyl sulfoxide
FTIR	Fourier-transform infrared spectroscopy
HEWL	Hen egg white lysozyme
HT	High-tension voltage in circular dichroism measurements
MRE	Mean residue ellipticity
OD	Optical density
ThT	Thioflavin T
Trp	Tryptophan
