# Supplementary material for: Influence of Urea and Dimethyl Sulfoxide on K-Peptide Fibrillation
Source: Int J Mol Sci. 2022 Mar 11;23(6):3027. doi: 10.3390/ijms23063027 (PMC8949822; doi:10.3390/ijms23063027)
Supplement: Supplementary file 1 [file ijms-23-03027-s001.zip › ijms-1614370-supplementary.pdf]

Supplementary materials to:

Influence of Urea and Dimethyl Sulfoxide on K-peptide Fibrillation

Jarosław Wawer, Jakub Karczewski, Robert Aranowski, Rafał Piątek, Danuta Augustin-Nowacka and Piotr Bruździak

Table S1. The ranges of high-tension (HT) voltage applied to the photomultiplier in circular dichroism measurements for analysed samples.

Solution composition	Incubation time [days]	HT range [V]
Water	0	321 V at 260 nm<br>606 V at 190 nm
Acetate buffer	0	321 V at 260 nm<br>616 V at 190 nm
Acetate buffer with dimethyl sulfoxide[1]	0	320 V at 260 nm<br>804 V at 215 nm
Acetate buffer with urea[2]	0	320 V at 260 nm<br>694 V at 197.2 nm<br>851 V at 195 nm
Water	14	320 V at 260 nm<br>558 V at 190 nm
Acetate buffer	14	319 V at 260 nm<br>556 V at 190 nm
Acetate buffer with dimethyl sulfoxide[1]	14	320 V at 260 nm<br>774 V at 212 nm
Acetate buffer with urea[2]	14	320 V at 260 nm<br>689 V at 195.4 nm<br>776 V at 194 nm

[1]high absorption of the sample does not permit to interpret the data
[2]high absorption at lower wavelengths allows only qualitative analysis

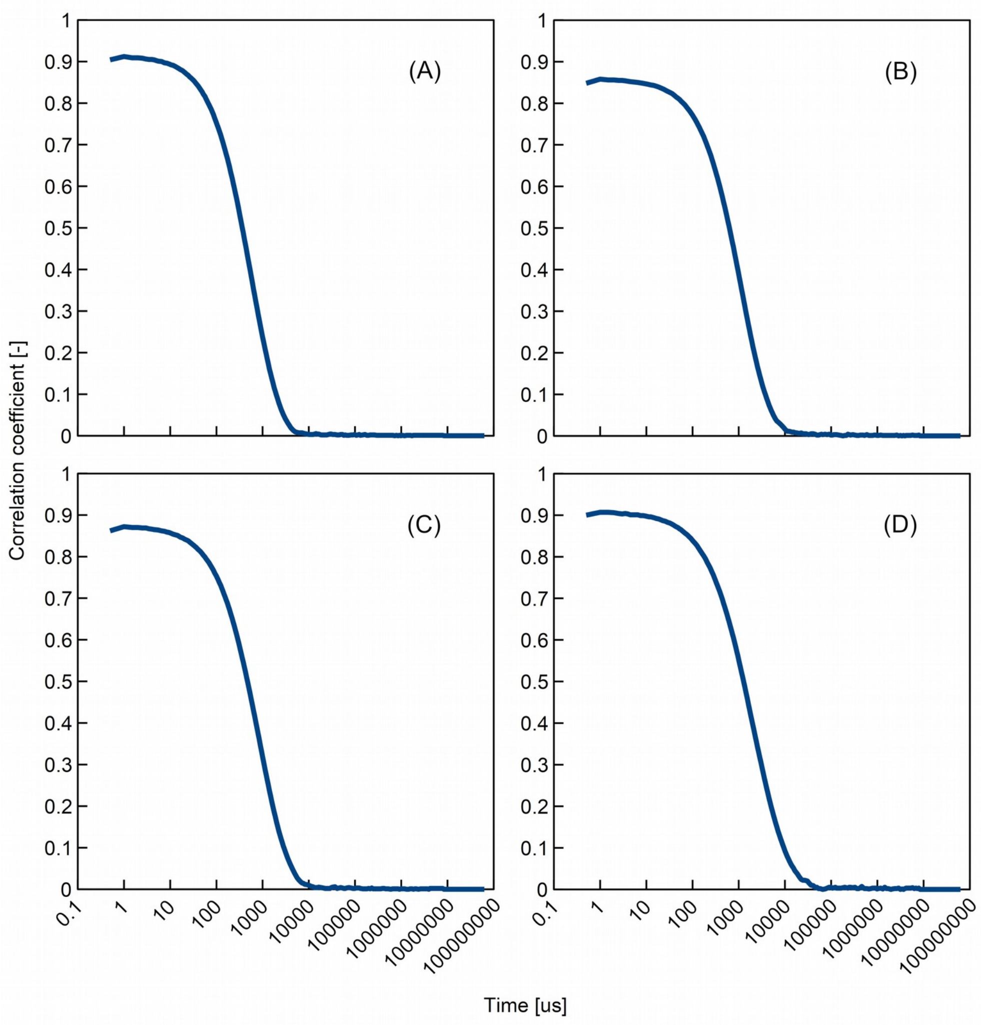

Figure S1 (part 1). The correlation functions recorded for dynamic light scattering measurements. The solutions contained K-peptide and: **(A,B)** water, **(C,D)** acetate buffer pH=4 without additives. Incubation time: left column 0 days, right column 14 days.

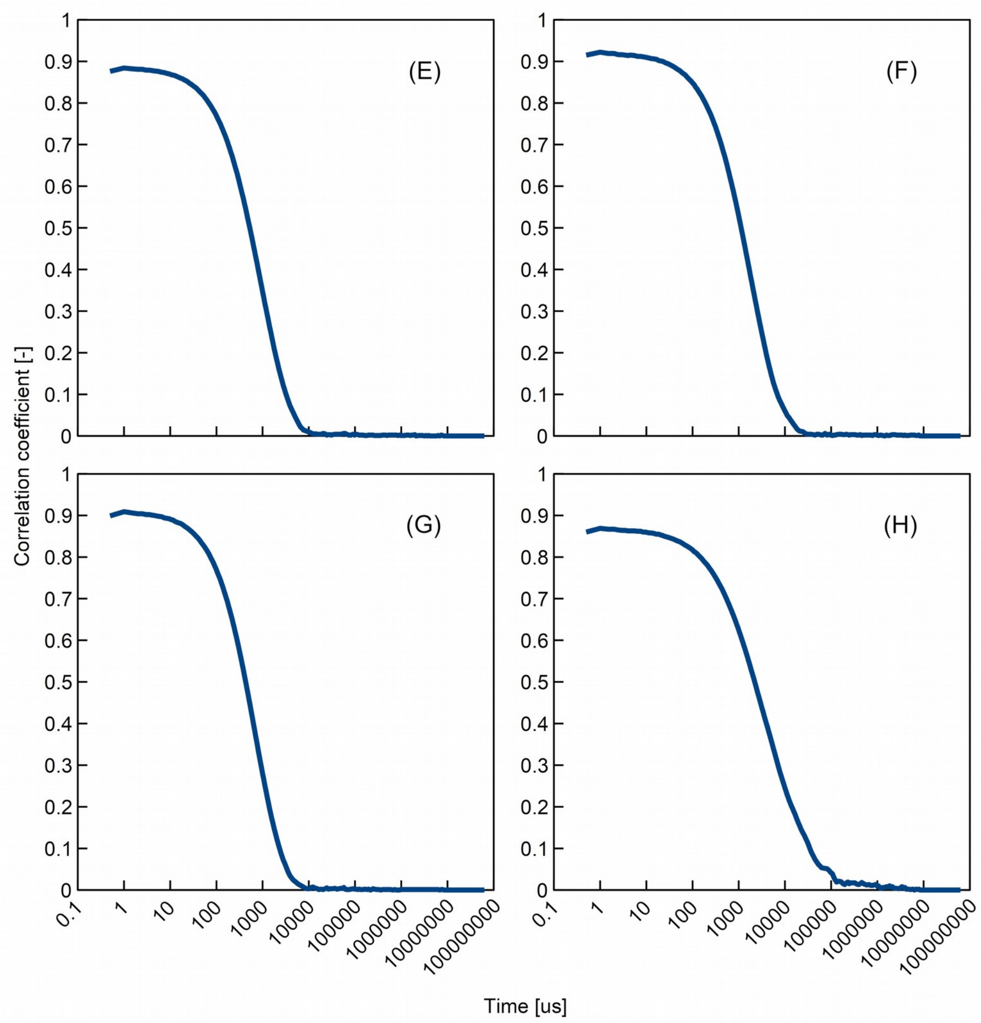

Figure S1 (part 2). The correlation functions recorded for dynamic light scattering measurements. The solutions contained K-peptide and: **(E,F)** acetate buffer with dimethyl sulfoxide, **(G,H)** acetate buffer with urea. Incubation time: left column 0 days, right column 14 days.

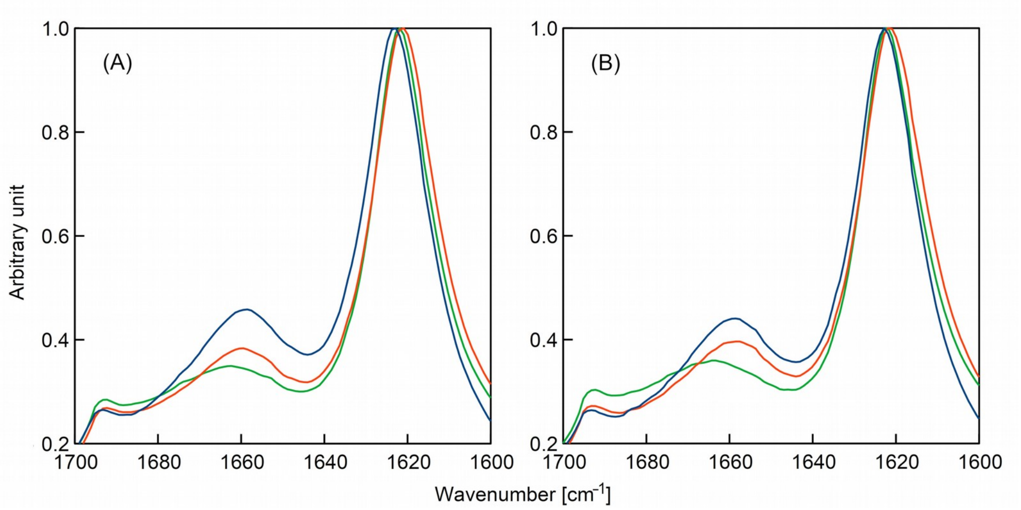

Figure S2. Normalised infrared spectra recorded for: **(A)** samples of non-incubated freshly prepared solutions of the K-peptide, **(B)** samples of the K-peptide incubated at 37 °C for 14 days. The peptide was dissolved in: water (blue line), acetate buffer pH=4 without additives (red line), acetate buffer with dimethyl sulfoxide (green line).

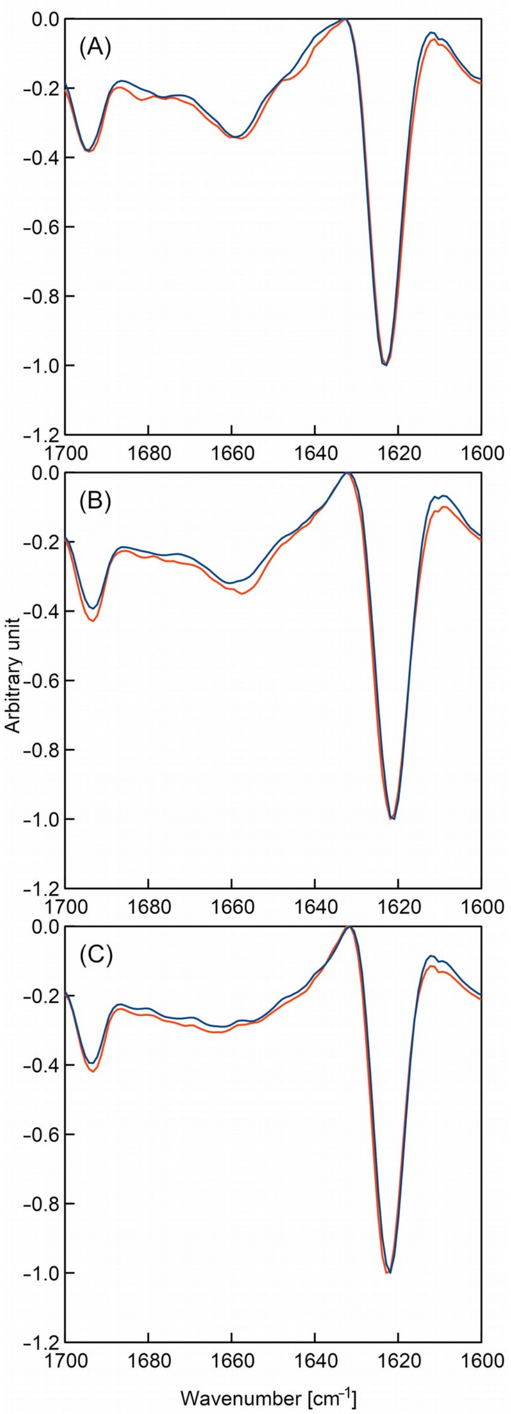

Figure S3. Infrared spectra—expressed as a second derivative of the absorbance—measurements for the samples of K-peptide in freshly prepared solutions (dark blue line) and for the samples of K-peptide incubated at 37 °C for 14 days (red line) dissolved in: **(A)** water, **(B)** acetate buffer pH=4 without additives, **(C)** acetate buffer with dimethyl sulfoxide.

Mass spectra and high performance liquid chromatography profile for K-peptide

Phone: +48 783 522 366
E-mail: info@peptideweb.com
Website: www.peptideweb.com

Bysewska 26b
80-298 Gdansk
Poland

Analiza chromatograficzna

Chromatographic analysis

RP-HPLC

Kolumna:	Waters, XBridgeShield RP 18, 3.5µm, 3x100 mm	Column
Faza ruchoma:	A – woda, B – acetonitryl; zawierające 0.1% TFA, v/v	Mobile phase A - water, B - acetonitrile, contains 0.1% TFA
Gradient:	30-100%, 10 min	
Przepływ:	1.0 ml/min	Flow
Detekcja:	UV, 214 nm	Detection

Chromatogram : GILQINSRW, 30-100% 10min 1mL 214nm1_channel1

System : HPLC
Method : 30-100%, 10min, 1mL, 214nm
User : User1

Acquired : 2018-07-16 14:47:17
Processed : 2018-07-26 11:25:58
Printed : 2018-07-26 11:26:03

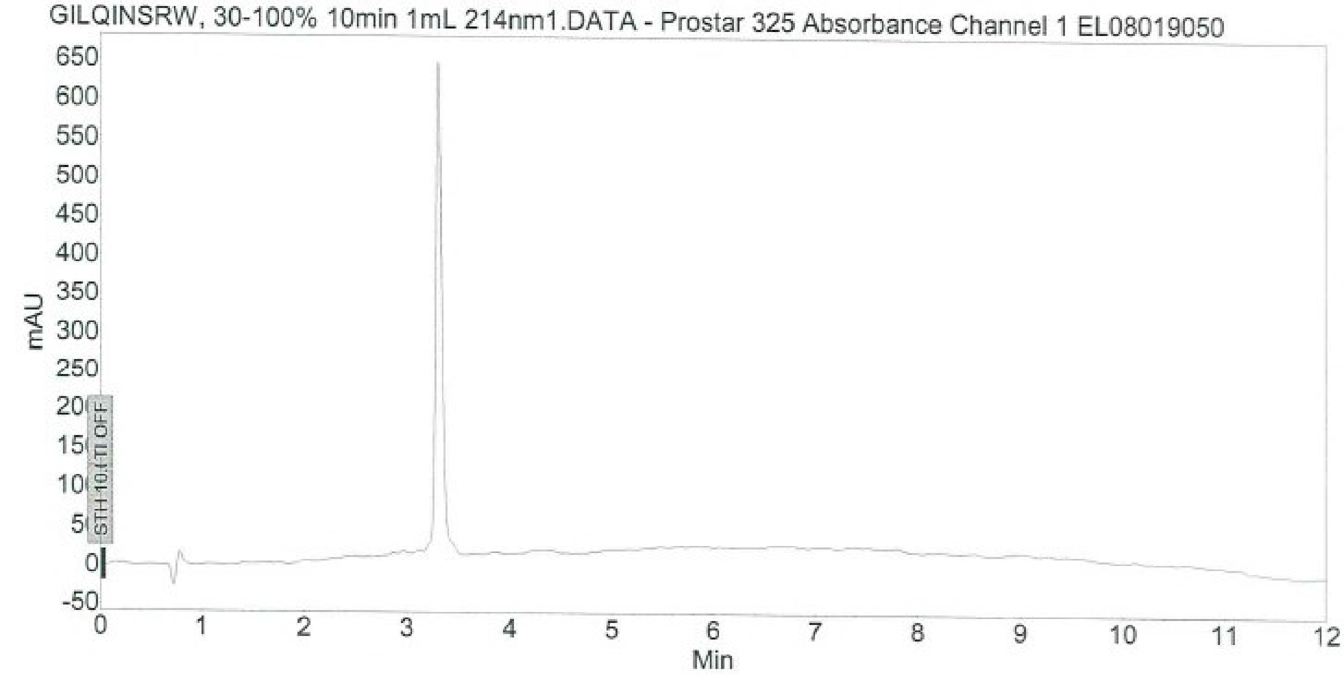

Peak results :

Index	Name	Time [Min]	Quantity [% Area]	Height [mAU]	Area [mAU.Min]	Area % [%]
Total			0.00	0.0	0.0	0.000

Phone: +48 783 522 366
E-mail: info@peptideweb.com
Website: www.peptideweb.com

Bysewska 26b
80-298 Gdansk
Poland

26-07-2018

Sekwencja aminokwasowa: GILQINSRW ← Amino acid sequence

Masa średnia: 1086,24 Da ← Average weight

Masa monoizotopowa: 1085,599 Da ← Monoisotope mass

Tryb jonizacji dodatniej ← Positive ionization mode

Zakres *m/z*: 100-1250 ← Range

z	Obliczone na podstawie masy średniej *m/z*	Obliczone na podstawie masy monoizotopowej *m/z*	Zmierzone *m/z*
1	1087,25	1086,61	1086,80
2	544,13	543,81	544,15

↑ Calculated on the basis of average weight

↑ Calculated from monoisotopic masses

↑ Measured

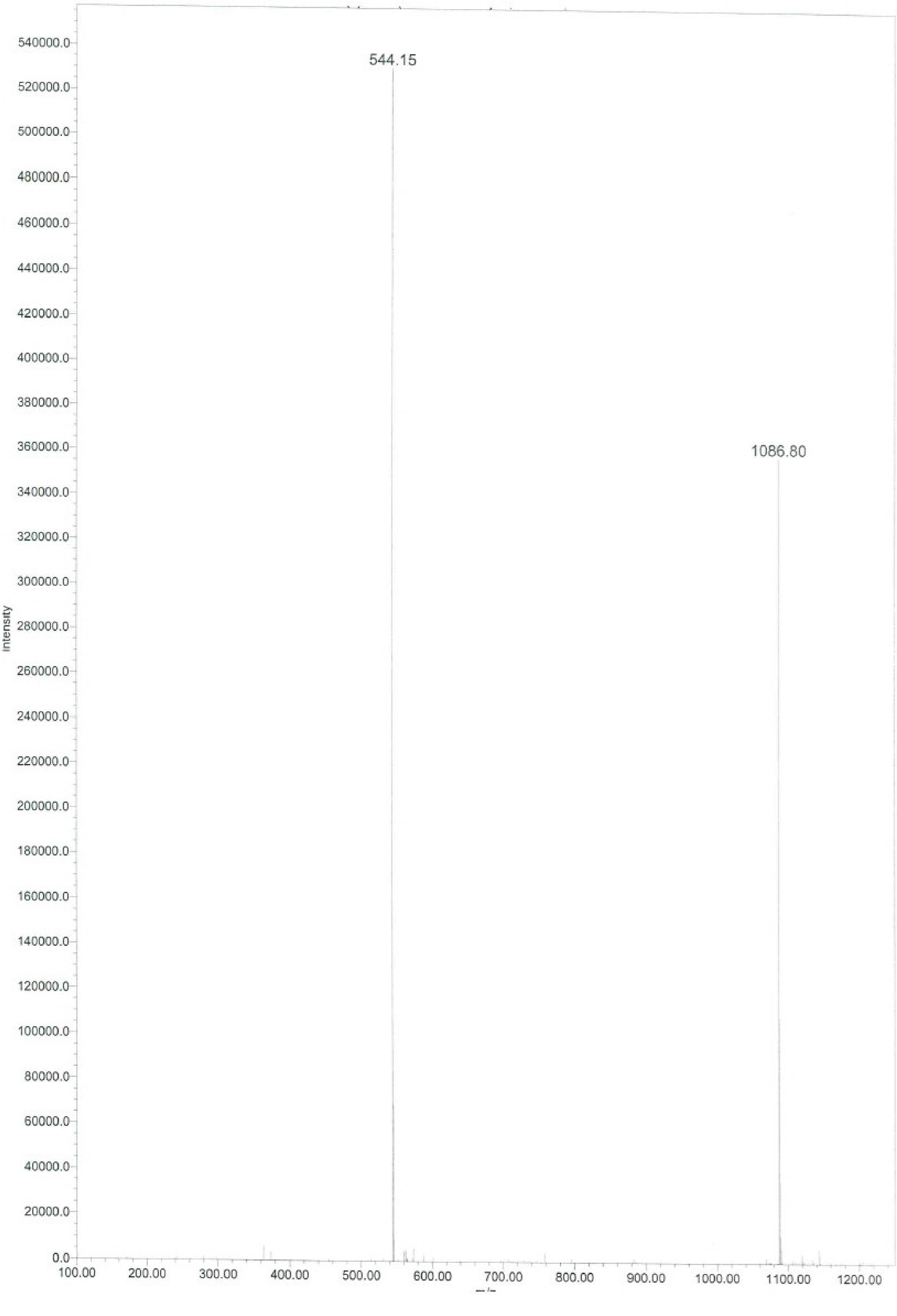

Intensity
544.15
1086.80
540000.0
520000.0
500000.0
480000.0
460000.0
440000.0
420000.0
400000.0
380000.0
360000.0
340000.0
320000.0
300000.0
280000.0
260000.0
240000.0
220000.0
200000.0
180000.0
160000.0
140000.0
120000.0
100000.0
80000.0
60000.0
40000.0
20000.0
0.0
100.00
200.00
300.00
400.00
500.00
600.00
700.00
800.00
900.00
1000.00
1100.00
1200.00
